# Strengths and weaknesses of guideline approaches to safeguard voluntary informed consent of patients within a dependent relationship

**DOI:** 10.1186/1741-7015-12-52

**Published:** 2014-03-24

**Authors:** Sara AS Dekking, Rieke van der Graaf, Johannes JM van Delden

**Affiliations:** 1 Julius Center for Health Sciences and Primary Care, University Medical Center Utrecht, Utrecht, The Netherlands

**Keywords:** Dependent relationships, Voluntary informed consent, Ethical guidelines, Clinical research, Research ethics, Double roles, Research nurses, Undue influence

## Abstract

**Background:**

It is thought that a dependent relationship between patients and physicians who enroll their own patients in research compromises voluntary informed consent. Therefore, several ethical guidelines for human subject research provide approaches to mitigate these compromises. Currently, these approaches have not been critically evaluated. In this article, we analyze the approaches of ethical guidelines to manage the influence of a dependent relationship between patients and physicians on voluntary informed consent and discuss the strengths and weaknesses of these approaches.

**Methods:**

We performed a review of international ethical guidance documents on human subject research, listed in the Oxford Textbook of Clinical Research Ethics and found through cross referencing. We also searched Global Ethics Observatory (GEObs) and the World Health Organization (WHO) website. Guidelines from all years were eligible for inclusion. The date last searched was December 2013.

**Discussion:**

We identified two basic guideline approaches: 1. a process approach, which focuses on the person who obtains informed consent, that is, an independent individual, such as a research nurse or counselor; and 2. a content approach, emphasizing the voluntary nature of participation. Both approaches are valuable, either because the influence of the physician may diminish or because it empowers patients to make voluntary decisions. However, the approaches also face challenges. First, research nurses are not always independent. Second, physician-investigators will be informed about decisions of their patients. Third, involvement of a counselor is sometimes unfeasible. Fourth, the right to withdraw may be difficult to act upon in a dependent relationship.

**Conclusions:**

Current guideline approaches to protect voluntary informed consent within a dependent relationship are suboptimal. To prevent compromises to voluntary informed consent, consent should not only be obtained by an independent individual, but this person should also emphasize the voluntary nature of participation. At the same time, dependency as such does not imply undue influence. Sometimes the physician may be best qualified to provide information, for example, for a very specialized study. Still, the research nurse should obtain informed consent. In addition, patients should be able to consult a counselor, who attends the informed consent discussions and is concerned with their interests. Finally, both physicians and research nurses should disclose research interests.

## Background

Ethical guidelines for human subject research assume that voluntariness of informed consent of patients for medical research could be compromised when their own treating physician obtains consent [1]. Guidelines are cautious with regard to dependent relationships between patients and physicians. When patients depend on physicians for care and treatment it is felt that patients may not feel free to refuse an invitation of their physician to take part in a study [2,3]. Patients may fear disappointing their physician or damaging the physician/patient relationship, which could influence their consent to research [4,5]. Several empirical studies have shown that treating physicians can have a considerable influence on the decision-making of their patients with regard to research [6-12]. Many ethical guidelines for human subject research have proposed strategies to safeguard voluntary informed consent of patients in the case of a dependent relationship [13-21].

In this article we analyze the approaches mentioned in the main ethical guidelines to safeguard voluntary informed consent in a dependent relationship and discuss the strengths and weaknesses of these approaches. Although some scholars have touched upon issues related to the guideline approaches, none of them has provided a systematic evaluation [3,22-25]. Ways to diminish threats to the voluntariness of informed consent deserve careful scrutiny, because of the widely acknowledged importance of voluntary consent [1]. In addition, implementing and executing those strategies will generally mean extra investments in terms of time and energy [1]. It is important to know whether this time is well spent.

## Methods

This article is reported according to the Methods section of the PRISMA checklist 2009.

### Protocol and registration

No review protocol exists for this study.

### Eligibility criteria

**Figure 1:**
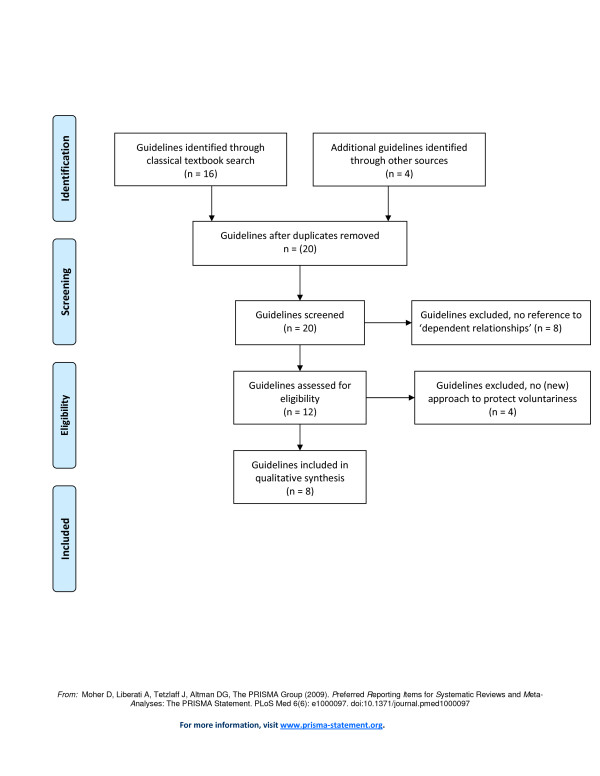
Flow diagram of the selection and inclusion of ethical guidelines.

We performed a review of ethical guidelines, reports and regulations on medical research with human subjects (henceforth referred to as ‘guidelines’). We only selected the main international guidelines and national guidelines that play a role in the international debate on medical research involving human beings. Further relevance of guidelines for our article was determined on the basis of the presence of phrases that concerned the relationship between patients and physicians in the context of medical research, and the potential undue influence of this relationship on patient decision-making with regard to research participation. First, guidelines that did not mention the potential influence of the relationship between patients and physicians on voluntary informed consent were excluded from our analysis and evaluation. Second, guidelines that only mentioned dependent relationships and its influence, but did not provide an approach to diminish this influence were excluded. Third, national guidelines that were not substantially different from international guidelines were excluded, because they would have no added value to our evaluation (see Figure 1).

Guidelines and regulations from all years were eligible for inclusion in our review, since also more historic regulations are generally considered to be of relevance for present-day analyses. Guidelines had to be written in the English language in order to be accessible to and relevant for an international audience.

### Information sources

We took the Oxford Textbook of Clinical Research Ethics [26] as a starting point. Furthermore, we searched the databases of PubMed and EMBASE to find literature to conduct our ethical evaluation. We also searched the Global Ethics Observatory (GEObs) database of UNESCO and the website of the World Health Organization (WHO). The date last searched for all these databases was December 2013. We had no specific dates of coverage, since we considered all possible publication dates of potential relevance.

### Search

We started with examining the main international ethical guidelines and national guidelines that play a role in the international debate on medical research involving human beings, based on the Oxford Textbook of Clinical Research Ethics which lists 16 ethical guidelines [26]. Of these ethical guidelines, eight did not mention the ethical issue of the influence of a dependent relationship between patients and physicians on voluntary informed consent and were excluded [27-35].

Two guidelines did mention that voluntary informed consent could be compromised within a dependent relationship, but they both did not provide an approach to safeguard voluntariness of patient consent and were therefore excluded [36,37]. Two guidelines both mentioned dependent relationships and an approach to protect voluntary informed consent [32,38]. However, since these guidelines have adopted the principles of the Declaration of Helsinki, they were not substantially different from this major international guideline and were excluded.

The four remaining guidelines of the Oxford Textbook of Clinical Research Ethics referred both to the influence of a dependent relationship on voluntary informed consent and suggested an approach to diminish this influence. Hence, these guidelines were included in our analysis and evaluation: The WMA’s *Declaration of Helsinki*[21]; the Council for International Organizations of Medical Sciences’ (CIOMS’) *International Ethical Guidelines for Biomedical Research Involving Human Subjects*[13]; the Canadian *Tri-Council Policy Statement, Ethical Conduct for Research Involving Humans*[20]; and the Australian National Health and Medical Research Council’s *National Statement on Ethical Conduct in Human Research*[14].

The second part of our search was based on the method of searching for titles added by cross referencing, since there is no one database in which all ethical guidelines are included. To find these additional guidelines, we searched the references of other guidelines and of articles discussing ethical guidelines for human subject research. This search provided us with four additional guidelines that conformed to our eligibility criteria: *Institutional Review Boards: Report and Recommendations* of the National Commission for the Protection of Human Subjects of Biomedical and Behavioral Research of the United States [18]; *Ethical Issues in Clinical Research in Neurology: Advancing Knowledge and Protecting Human Research Subjects* of the Ethics and Humanities Subcommittee of the American Academy of Neurology (in neurology research and care are often combined, meaning that patients are frequently recruited within a dependent relationship) [17]; *the Ethics Manual, Sixth Edition* of the American College of Physicians [19]; and *Managing Conflicts of Interest in the Conduct of Clinical Trials* of the American Medical Association’s Council on Ethical and Judicial Affairs [15].

To find further internationally important guidelines we performed a search in ‘Database 4: Ethics Related Legislation and Guidelines’ of the GEObs database of UNESCO. The theme we searched within this website was ‘Medical research with human beings’ and we included all legal categories (that is, treaties, constitution, domestic laws, authoritative case laws and guidelines). Finally, we searched the website of the WHO. Both the database and the website did not provide us with additional ethical guidelines that were of relevance for the international debate on voluntary informed consent and dependent relationships between patients and physicians.

In order to find (empirical) support for the conduct of our evaluation of the guideline strategies, we performed several searches in PubMed and EMBASE, using various combinations of the following search terms: ‘dependent relationships’, ‘dependency’, ‘physician-patient relationship’, ‘clinician-patient relationship’, ‘medical research’, ‘clinical research’, ‘research’, ‘clinical trials’, ‘ethical guidelines’, ‘guidelines’, ‘policy’, ‘ethical guidance’, ‘voluntariness’, ‘voluntary informed consent’, ‘informed consent’, ‘consent’, ‘influence’, ‘empirical’, ‘qualitative’ and ‘quantitative’.

### Data collection process

All included guidelines were examined for an explicit description of an approach to protect voluntary informed consent for research when it is obtained within a dependent relationship between patients and physicians.

### Synthesis of results

We selected the phrases of the eight included guidelines that expressed an approach to diminish the risk of compromised voluntariness due to the undue influence of the treating relationship with and dependence upon the physician. We derived the key aspects of each of these guideline phrases and compared them in order to infer whether they contained similar approaches to safeguarding voluntary informed consent. We identified two basic approaches within these ethical guidelines, that is, a process versus a content focused approach.

With regard to the evaluation of the two guideline approaches, we used empirical data from the studies that showed up in our PubMed and EMBASE searches. In addition, where empirical support was lacking our evaluation is based on rational argumentation applied to the particularities of the clinical research context.

## Discussion

### Defining dependent relationships, voluntary informed consent and vulnerability

#### *Dependent relationships*

Dependency is often mentioned as a critical feature of physician-patient relationships in clinical research, both in the ethical guidelines for medical research with human beings [13,14,20,21] and in literature [39,40]. A dependent relationship in the context of medical research can be defined as a pre-existing treating relationship between patients who are prospective participants and their physicians which carries the potential for undue influence due to the dependence of patients upon their physicians for care and treatment [13-15,17,18,20,21]. Although some national guidelines and legislations acknowledge that also in the clinical context undue influence by the physician should be avoided [41-43], we will focus on the context of medical research.

#### *Voluntary informed consent*

All reviewed guidelines express the thought that dependent relationships should be considered carefully, due to the influence the treating physician can have on the consent of the prospective participant. Although this influence is not necessarily regarded as problematic, the potential for undue influence frequently is emphasized. The reason that undue influence is regarded undesirable is because it compromises voluntary informed consent. This is reflected in the definition of the Belmont Report, which states that voluntary informed consent ‘requires conditions free of coercion and undue influence’ [28]. Two recently suggested interpretations of voluntary informed consent also regard the concept of influence as key to their definition of voluntary informed consent, although they speak of ‘illegitimate’ [24] and ‘controlling’ [44] influences, respectively.

Our working definition of voluntary informed consent is the one provided by the Belmont Report [28], which has also been accepted by the majority of the guidelines from our analysis [13,14,17,18,20,21]. With this definition as a starting point, we are able to evaluate the guideline approaches according to their capacity to diminish the undue influence of physicians on patients when they recruit them for participation in medical research.

#### *Vulnerability*

To understand why patients recruited by their own physician are in need of protection by ethical guidelines, we need to consider the concept of vulnerability. Three recent accounts of vulnerability acknowledge the importance of the situation or context of research participants for attributing vulnerability [45-47]. Based on these accounts we suggest that the context of a dependent relationship could add an extra layer [47] or increased likelihood of vulnerability on patients who are recruited for research, due to the hierarchical structure [45] that is present in a dependent relationship. Vulnerability then can be seen as a claim to special protection [45], which strengthens the assumption that additional safeguards for patients recruited by their own physician are sensible and deserve careful scrutiny.

For now, we consider that dependent relationships are generally regarded as a potential source of undue influence, that undue influences compromise voluntary informed consent, and that a dependent relationship may render patients vulnerable and, therefore, in need of special protection.

### Two basic guideline approaches

It appears that ethical guidelines for medical research with human beings suggest two different approaches to manage the impact of a dependent relationship on voluntary informed consent. The first approach focuses on the process of obtaining informed consent; the second approach focuses on the content of the information that is communicated to the patient (see Table 1). Most guidelines refer to only one of the two approaches [15,17-21], two guidelines include both approaches [13,14].

**Table 1:** Guideline approaches to protect voluntary informed consent within a dependent relationship

**Guidelines**	**Professional association**	**Approach**
Declaration of Helsinki, 2013	World Medical Association	§27: ‘When seeking informed consent for participation in a research study the physician must be particularly cautious if the potential subject is in a dependent relationship with the physician or may consent under duress. In such situations the informed consent must be sought by an appropriately qualified individual who is completely independent of this relationship.’
International Ethical Guidelines for Biomedical Research Involving Human Subjects, 2002	Council for International Organizations of Medical Sciences (CIOMS) in collaboration with the World Health Organization (WHO)	Commentary on Guideline 6: ‘The physician/investigator must assure [patients] that their decision on whether to participate will not affect the therapeutic relationship or other benefits to which they are entitled. In this situation the ethical review committee should consider whether a neutral third party should seek informed consent.’
Tri-Council Policy Statement, Ethical Conduct for Research Involving Humans, 2010	Canadian Institutes of Health Research, Natural Sciences and Engineering Research Council of Canada, and Social Sciences and Humanities Research Council of Canada	Article 3.1 a (Application): ‘Pre-existing entitlements to care, education and other services should not be prejudiced by the decision of whether or not to participate in, or to withdraw from, a research project. Accordingly… a physician should ensure that continued clinical care is not linked to research participation.’
National Statement on Ethical Conduct in Human Research, 2007	National Health and Medical Research Council, Australia	4.3.2 ‘In the consent process, researchers should wherever possible invite potential participants to discuss their participation with someone who is able to support them in making their decision.’
		4.3.10 ‘Where the researcher has a pre-existing relationship with potential participants, it may be appropriate for their consent to be sought by an independent person.’
Institutional Review Boards: Report and Recommendations, 1978	The National Commission for the Protection of Human Subjects of Biomedical and Behavioral Research, United States	‘In cases in which the investigator is responsible for the care of the subjects, the IRB may require that a neutral person, not otherwise associated with research or the investigator, be present when consent is sought, to explain the research to prospective subjects, or to observe the conduct of the research … Such a person may be designated to play a role in informing subjects of their rights and the details of protocols, assuring that there is continuing assent to participation, determining the advisability of continued participation, receiving complaints from subjects, and bringing grievances to the attention of the IRB as part of its continuing review of research.’
Ethical issues in clinical research in neurology: advancing knowledge and protecting human research subjects, 1998	The Ethics and Humanities Subcommittee of the American Academy of Neurology, United States	‘[R]esearchers and IRBs may want to consider additional safeguards. For example, the IRB may request that an “uninterested” individual … discuss with prospective subjects the research study and other clinical or research alternatives.’
Ethics Manual, Sixth Edition, 2012	Ethics, Professionalism, and Human Rights Committee, American College of Physicians, United States	‘It should … be clear to patients that participation in research is voluntary and not a requirement for continued clinical care. The right to withdraw consent and discontinue participation at any time must be communicated.’
Managing Conflicts of Interest in the Conduct of Clinical Trials, 2002	The council on ethical and judicial affairs, American Medical Association, United States	‘[T]he physician who has treated a patient on an ongoing basis should not be responsible for obtaining that patient’s informed consent to participate in a trial that will be conducted by the physician. .. Instead … someone other than the treating physician should obtain the participant’s consent. The non-treating health care professional also could remain available to answer additional questions during the trial.’

#### *Process approach*

The informed consent process concerns two primary activities: to inform patients of the study details and to obtain written informed consent [21]. The first guideline approach to diminish the influence of the dependent relationship on patients is to have an independent, qualified individual take over one or both of these two primary tasks of the physician-investigator [13-15,17,18,21]. First, this independent individual can carry out the informed consent procedure instead of the physician-investigator [13-15,21], to separate the informed consent process from the therapeutic relationship [4]: ‘Where the researcher has a pre-existing relationship with potential participants, it may be appropriate for their consent to be sought by an independent person’ [14]. This person, who could be a research nurse or any other health care professional not directly responsible for the usual care of the patient in question, should be ‘completely independent’ [21] from the treating relationship patients have with their physician.

Second, the independent individual could explain the study details and possible alternatives to research participation to the patients-subjects and answer the questions patients have with regards to the provided information [14,15,17,18]. When fulfilling these tasks, the independent individual functions as a kind of counselor.

#### *Content approach*

The second guideline approach aims to safeguard voluntary informed consent within a dependent relationship by demanding that certain vital pieces of information are conveyed to patients. One guideline stresses that ‘the right to withdraw consent and discontinue participation at any time must be communicated’ [19]. One reason why patients find it difficult to refuse participation in the study is because they fear their refusal will adversely affect the care they receive or the relationship with their physician [48]. Therefore, several guidelines [13,19,20] require from physicians that they emphasize to their patients that they will not suffer any negative consequences from possible refusal or withdrawal from the study.

### Strengths and weaknesses of both approaches

#### *Process approach*

Some positive aspects of what we have called the process approach have been suggested in the literature. By conveying the task of obtaining informed consent to an independent individual, the informed consent procedure is made more formal and clearly distinct from the practice of caregiving [22,49]. The physician could thereby communicate an explicit message to the patient, emphasizing the difference between research and usual practice [3,22].

To delegate the informed consent process to an independent individual is important in order to avoid patients feeling intimidated by their physician [25]. It is mentioned that research nurses (or other study coordinators) are considered to be more neutral than physicians and researchers, making them better suited to obtain patient informed consent [11] since they are not directly involved with the usual care of the patients [49]. Their neutrality arises because they are independent from the treating relationship between patients and physicians. Since guidelines regard dependent relationships as problematic, this initial neutrality of the research nurse is thought to diminish the potential undue influence of the physician on patient decision-making [2,49].

However, considerable demands are often placed upon research nurses to recruit and retain a high number of patients to meet the study targets [50,51], and sometimes their employment depends upon their succeeding to complete trials in order to gain funding for subsequent studies. Therefore, it is questionable how neutral the position of research nurses actually is.

In addition, research nurses are frequently not as independent from the treating relationship with patients as is suggested. Although there is no generally accepted, standardized description of the tasks of the clinical research nurse [52], an important aspect of their role is that in addition to their research related tasks, they are usually also involved in the care of the patients they have recruited for research [51]. Clinical research nurses monitor the participating patients, explaining additional details, providing support and carrying out the distribution of medication [53,54]. Therefore, they must find a balance between the needs of patients and the demands of the research protocol [55]. Qualitative studies have shown that research nurses themselves experience such a role conflict [50,51,54], although they generally feel that their role as patient advocate is their primary one [54].

The caregiving role of research nurses entails ongoing interaction with patients, which could result in a relationship between research nurses and the patients they take care of [50,54], similar to the dependent relationship between patients and physicians. A risk of such a relationship is that patients feel they are in some way dependent upon the research nurse, leading patients to think they should follow the nurse’s recommendations [54]. This could in turn prevent them from withdrawing from the study, which is contrary to the demand that voluntary informed consent needs to be protected both at the start of the study and as the study progresses [1].

In addition to factors specifically applying to the research nurse, several other challenges to the process approach can be assumed. The strategy to have an independent individual obtain informed consent instead of the treating physician starts from the assumption that such a person makes it easier for patients to refuse. However, two aspects of clinical research practice should be considered to assess the potential effectiveness of having a third person obtain consent.

First, the independent person is usually invited by the physician-investigator or appointed by the hospital where the patient is treated [23]. As a result, patients may not perceive this supposedly independent person as truly independent from the relationship they have with their physician. Therefore, this person could just as well unduly influence the consent of the prospective participant.

Second, even though an independent individual obtains the informed consent of patients, several situations exist in which the physician of these patients will also be notified of their decision. When patients express their refusal to the independent individual, their own physician who conducts the study in question will typically be informed of this decision [23] since he or she needs to know whether the patient participates in the study or should receive standard treatment. In a dependent situation the treating physician will always know whether the patient declined or accepted research participation, because this physician is the one who both conducts the study and has the responsibility for the treatment of the patient. So, the influence of the dependent relationship is not removed, meaning that some patients still consent against their wishes because their physician will know anyhow.

Furthermore, even when the treating physician is not involved with the study the patient is included in, it will in some situations still be necessary to inform the physician of the decision of their patient. For instance, in case research participation influences the usual care or when during the trial clinical problems arise that should be dealt with by the physician [23]. Thus, the decision of whether a participant has given consent is always disclosed, irrespective of possible blinding of the physician for the study drug that a patient will receive if the patient has given consent. It is the disclosure of the decision to participate that is relevant in light of threats to voluntariness.

The use of counselors to support patients in decision-making and empower patients to choose their preferred option seems a useful way to improve voluntary consent [54], since they should be able to ask relevant questions and provide support to patients [25]. However, a challenge for this approach emerges when counselors lack medical expertise [25]. This will especially be the case if the counselor is a friend or relative [23].

Another challenge to the involvement of counselors is mainly practical. It will be difficult for many hospitals or other health care facilities to train and appoint a sufficient number of counselors to provide support for the large number of patients that is recruited within a dependent relationship.

Support of counselors or of other kinds of representatives is not unique for the context of dependent relationships. Several ethical guidelines also propose advocates or counselors for patients with insufficient mental capacity to consent on their own [13,14,20,21]. A factor that is often mentioned is that these advocates should consider the interests and preferences of the prospective participant in question [14,20].

Interestingly, in describing the role of the counselor who supports patients in dependent relationships, ethical guidelines merely state that these counselors should provide study details, be available for questions during and after the informed consent procedure and assess the advisability of further participation. As such, the guidelines do not explicitly mention that counselors should take the interests and preferences of patients into account.

#### *Content approach*

The rights to withdraw and refuse and the absence of negative consequences should also always be communicated to patients recruited outside the context of a dependent relationship to emphasize the voluntary nature of research participation [21,35]. Without this information, not all patients will be aware of their rights to refuse and withdraw without any retribution, which would be an ethically undesirable situation and would mean an infringement upon the validity of informed consent [56].

However, the positive effects of the content approach on patients in a dependent relationship have hardly been studied. We think that this results from the content approach not previously being identified as one of two approaches to protect voluntary informed consent in a dependent relationship: most scholars and studies focus on introducing an independent individual in the informed consent procedure and more specifically on the role of the research nurse.

Therefore, advantages of the content approach are not readily supported by the literature. Although one study shows that some data managers (for example, research nurses, research assistants, study coordinators) feel that presenting information in a non-coercive manner and making patients aware of their rights to refuse and withdraw could ensure voluntary participation [25], it is unclear how patients perceive this. Arguably, conversations with a research nurse or some other member of the research team could provide patients with robust knowledge [57], which could aid them with their decision-making. Yet, at least two challenges remain for the content approach.

First, statements on the right to withdraw and the absence of negative consequences can easily become void expressions rather than effective ways to protect the voluntary informed consent. The disclosure of information is not sufficient to meet the requirements for informed consent [25], since patients should also understand the information they receive [58]. The manner in which the physician communicates the information is of great importance for patient comprehension [58], something that ethical guidelines currently do not incorporate.

Second, although a review by Mandava *et al*. has shown that at least 75% of research participants are generally aware of their right to withdraw [59], in certain contexts a gap exists between knowing something and acting upon it [60]. Understanding information on voluntariness does not equal voluntary informed consent. A recent study by Horwitz *et al*. tried to untangle comprehension and voluntariness in the informed consent process for a HIV trial in Haiti [61]. They found that even though the participants showed good understanding of the study details, including the voluntary nature of the study, 11% gave responses suggesting involuntary consent. One of these responses concerned the belief ‘that a “volunteer” is someone who makes an irreversible commitment to remain in the study’ [61].

This study shows that even patients who had understood the provided information did not realize that they were indeed free to withdraw at any moment. For patients within a dependent relationship, the gap between knowing and acting upon that knowledge may be even greater, since patients depend upon their physician for the provision of care and treatment. Thus, although patients know and understand their rights, they could be hesitant to withdraw from the study even though they know they have the right to, since they do not want to disappoint their treating physician [22].

### Moving forward

The approaches as suggested by ethical guidelines seem suboptimal safeguards with respect to the voluntariness of informed consent in a dependent relationship. First, the influence of the physician is not necessarily sufficiently diminished when someone else obtains informed consent. Second, the right to withdraw cannot sufficiently be protected by simply pointing at this right and this right may be difficult to act upon in a dependent relationship.

It is time that physicians, investigators and members of Research Ethics Committees acknowledge that current guideline approaches do not appropriately protect patients who are enrolled by their own physician. Moreover, we believe that quick and easy solutions to the problem of compromised voluntariness do not exist. At least, the two existing approaches should be combined. The content approach, although on its own not sufficient, is of pivotal importance. Patients should always be informed about the voluntary nature of research participation and about the absence of negative consequences when they refuse or withdraw. And as regards the process approach, it is not sufficient if obtaining informed consent is delegated to a presumed neutral third party, since these persons are often not independent, both of the treating relationship and of the study in question. At the same time, feasible inclusion by the research nurse or an equally qualified person is preferred over inclusion by the treating physician, since this physician is the primary caregiver.

However, a dependent relationship does not always imply compromised voluntariness of informed consent of the patients who are recruited for research by their own physician. People are influenced by others all the time and not all influences necessarily pose a threat to voluntary informed consent [24,44]. Consequently, being in a dependent relationship does not imply that patients should make decisions about research participation completely independent from the physician. Moreover, in some dependent situations it can be preferable that the physician provided the patient with information instead of the research nurse, for instance, if the research is the means through which treatment is delivered or if a study is too detailed and specialized for research nurses to explain. The informed consent can then be signed in the presence of a research nurse or an equally qualified colleague.

In the clinical context the relevance of social relationships for decision-making has already been recognized, since ‘preferences developed independently are not necessarily better than treatment preferences developed in collaboration’ [62]. One could also argue that in the context of medical research, patients appreciate being informed by their own physician, if patients feel they know and trust this person and feel comfortable speaking to their physician. For instance, a review by McCann *et al*. has shown that patients consider the interaction with their physician as key to their research involvement [63], indicating that a supportive and active role with their physician is something that patients find valuable when deciding about research.

Although caution in the case of a dependent relationship is still required, an existing dependent relationship should not by definition prevent a supportive and engaged role of physicians, as long as they are aware of their potential influence and ‘recognize how their interactions and relationships with patients can either enable or impair patients’ autonomy’ [64]. So, the challenge for physicians is to be engaged and supportive, without unduly influencing their patients. If physicians approach their own patients for research they should find a balance between undue influence at one end of a continuum and independent decision-making on the other end. They should be honest and transparent about their research related interests and should give responsive support adapted to individual patients and their needs, preferences and abilities [62]. These requirements equally apply to research nurses who cannot be independent.

Where feasible, patients should be able to ask for additional protection by involving a counselor. This is true also in case it is the research nurse who obtains informed consent. Although some guidelines suggest that patients should be in the position to discuss research participation and the study details with someone else [14,15,17,18], the guidelines do not elaborate on what his or her role exactly is. We suggest that this counselor should be independent (for example, a regular nurse who works in a different department and is not involved in the study), specifically educated and be able to support patients in their decision-making. Therefore, on the patient’s request, this counselor should be present during informed consent discussions. He or she should pay close attention to the preferences and views of patients in order to enable them to make decisions that fit their goals and values. Also, the counselor should be available to answer any questions patients might have afterwards or during the proceedings of the trial.

### Further research

In order to effectively reflect on the interaction between patients and their physicians during the informed consent procedure and develop an approach that respects medical research practice, more research is needed. First, we need to know whether patients indeed feel pressure to accept an invitation for research from their physician due to the prior therapeutic relationship. Second, if voluntary informed consent is compromised when obtained within a dependent relationship, it should be investigated how extensive the problem is and which factors contribute to it. Factors that could be of importance are whether physicians recruit patients for their own research or for someone else’s [15,17]. Furthermore, the degree of dependency [20] can be of importance, which in turn can be influenced by the severity of the disease [40] and the length of the treating relationship [17]. Third, the merits of our new proposal, that is, a combination of the process and content approaches complemented by an upgraded version of the counselor, should be empirically studied, taking the perspectives and experiences of patients and physicians into account.

## Conclusions

Ethical guidelines try to manage the impact of a dependent relationship on voluntary informed consent in two ways. One approach focuses on the process of obtaining informed consent; the other on the content of the information that is communicated to the patient. Some guidelines include both approaches; other guidelines only articulate one of them. Our analysis shows that although both approaches could have some favorable impact on voluntary informed consent of patients, they also face challenges. Research nurses are not independent of the treating relationship patients have with their physician, since they frequently provide care to the included patients. Also, they are not neutral with regard to the study they recruit patients for, as their job often depends on assuring high inclusion rates. Any other health care professional is likely to be regarded by patients as belonging to the team of the physician, which could also influence voluntary informed consent.

Furthermore, even if patients express their refusal to an independent individual, in many instances their own physician will be informed of this decision, which means that the influence of the physician on voluntary informed consent is still present. Making patients aware of their rights to refuse and withdraw at any time is important, but might not be convincing for patients enrolled by their own physician, because they depend on the physician for care and treatment.

At least the process and content approach should be combined. Patients in a dependent relationship should in all instances be informed of their rights to withdraw and refuse without any negative consequences with regard to their care or the relationship with their physician. Furthermore, inclusion by a research nurse is preferred over inclusion by the physician, since the physician is the primary caregiver of the patients. Deviations from this rule are conceivable in cases where research is part of the treatment or where a physician will be better able to explain the research protocol than the research nurse. After all, dependency as such does not imply undue influence. Patients need not make completely independent decisions in order for these decisions to be voluntary. In such cases, it is important that physicians are aware of their own influence and be transparent and honest about their existing research interests. This is also the case for research nurses who cannot be independent.

To further prevent undue influence, patients should be able to ask for a specifically educated and independent counselor, who can attend the informed consent process. This should also be the case if the research nurse obtains informed consent. These counselors should be in the position to provide support to patients and optimally safeguard voluntary informed consent, if they actively take the values and preferences of patients into account.

## Competing interests

This article is part of a larger research project which is funded by the Dutch grant supplier ZonMw, grant number 113203201. The views expressed here do not represent the position of ZonMw. The authors declare that they have no competing interests.

## Authors’ contributions

We can confirm that all three authors contributed substantially to the conception and development of the article. SD wrote the initial draft of the article. All authors contributed to its revision and approved the final version of the manuscript submitted for publication.
